# Exploring the roles of conserved context‐dependent cis‐regulatory elements (cdCREs) in multicellularity, human health and disease

**DOI:** 10.1111/febs.70174

**Published:** 2025-07-04

**Authors:** Andrew McEwan, Alexander Rattray, Greg Hutchings, Elizabeth Hay, Chris Murgatroyd, Alasdair MacKenzie

**Affiliations:** ^1^ Institute of Medical Sciences, Foresterhill University of Aberdeen UK; ^2^ John Dalton Building, Manchester Metropolitan University UK

**Keywords:** cis‐regulatory element, comparative genomics, context‐dependent, CRISPR genome editing, DNA methylation, gene regulation, *in vivo* models, multicellular

## Abstract

Human development and health depend on the precise expression of relevant genes in specific cells, at precise times and in response to appropriate stimuli. This is known as context‐dependent gene regulation and relies on the activities of a diverse ‘zoo’ of DNA elements within the genome that are collectively called context‐dependent cis‐regulatory elements (cdCREs). cdCREs may comprise as much as 10% of the genome and include better‐known sequences such as enhancers, silencers and promoters that form the basis of complex multicellularity. Diverse vertebrate body plans not only share considerable phenotypic similarities but also cell types, the genes they express and, in a growing number of cases, in the function and nucleotide sequence of cdCREs. The current review will critically evaluate current methodologies to identify cdCREs and re‐evaluate a place for comparative genomics amongst them. We will also explore the function of cdCREs and discuss methods of analysing their function in disease‐associated physiologies and behaviours using *in vivo* models such as CRISPR‐generated GA mouse lines. Finally, we will study the effects of epigenetic mechanisms such as DNA methylation on cdCRE activity and examine how genetics and epigenetics can interact to alter disease susceptibility. Given that genome‐wide association studies (GWAS) suggest that 95% of disease‐associated genomic variation reside in the 98% of the less understood noncoding genome, the need to understand the role of conserved vertebrate cdCREs in development and health *in vivo* has never been more pressing.

## Graphical abstract

Conserved context‐dependent cis‐regulatory elements act as a major reservoir of disease‐associated polymorphisms in the human genome and are also affected by epigenetic change. This ‘state‐of‐the‐art’ review explores the latest developments in studying these enigmatic elements and how the effects of disease‐associated polymorphisms and environmentally induced DNA methylation changes can be characterised *in vivo* thanks to advances in genome editing technologies, behavioural analysis and next‐generation sequencing.

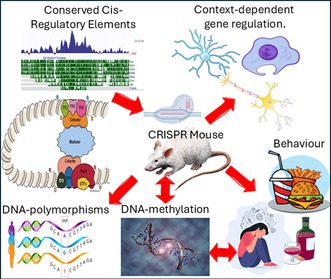

**Abbreviations**
5hmC: 5‐hydroxymethylcytosine
5mC: 5‐methyl cytosine
A: adenine
AAV6: adenovirus‐associated virus 6
ATAC‐seq: assay for transposase‐accessible chromatin using sequencing
AVP: arginine vasopressin
BDNF: brain‐derived neurotrophic factor
BDNF‐AS: BDNF‐antisense gene
BE5.1: a context‐specific cis‐regulatory element influencing BDNF expression
BP4: BDNF promoter 4
Cas9: CRISPR‐associated protein 9
cdCRE: context‐dependant cis‐regulatory element
ChIP: chromatin immunoprecipitation
CpG: cytosine‐phosphate‐guanine di‐nucleotide
CRE: Cis‐regulatory element
CRISPR: clustered regularly interspaced short palindromic repeats
CTCF: CCCTC‐binding factor
DNA: deoxyribonucleic acid
ELS: early life stress
EMSA: electrophoretic mobility shift assay
ENCODE: Encyclopaedia of DNA elements
GA: genetically altered
GAL: gene encoding the galanin neuropeptide
GAL5.1: a context‐specific cis‐regulatory element driving GAL expression
GFP: Jellyfish gene encoding the green fluorescent protein
GWAS: genome‐wide association study
H3K27ac: monoacetylation of lysine 27 of histone 3
H3K4me1: monomethylation of lysine 4 of histone 3
HDR: homology‐directed repair
Hi‐C: Hi‐C chromatin conformation capture
Hox: Homeobox containing homeotic gene
IL‐12: interleukin 12
IL‐23: interleukin 23
LacZ: bacterial gene encoding the β‐galactosidase protein
LD: linkage disequilibrium
Mb: megabase (1 × 10^6^ base pairs)
Mecp2: methyl‐CpG‐binding protein 2
NGS: next‐generation sequencing
NHEJ: nonhomologous end joining
PAM: protospacer adjacent motif
PVN: Paraventricular nucleus of the hypothalamus
QPCR: quantitative polymerase chain reaction
RNP: ribonucleoprotein
scRNA‐seq: single‐cell ribonucleic acid sequencing
sgRNA: single guide ribonucleic acid
SHH: Sonic hedgehog
SNP: single nucleotide polymorphism
SON: supraoptic nucleus of hypothalamus
T: thymidine
TAD: topologically associating domains
TET: Ten‐Eleven Translocation dioxygenase enzymes
TF: transcription factor
TSS: transcriptional start site
WGS: whole genome sequencing

## Introduction

For the vast majority of the 3.6–4 billion years that life has existed, it took the form of single‐cell organisms [1]. There is evidence to suggest that the first multicellular animals, also known as metazoans, arose in the early Ediacaran period (609 My) [2]. Whilst early multicellular life forms, such as sponges and slime moulds, could be regarded as loosely multicellular, later metazoans such as the bilateria, that include arthropods and vertebrates, developed more structured and highly complex body plans during the Cambrian period. This allowed the perception and directional movement required for sophisticated behaviours such as food acquisition, reproductive behaviours [3] and predator–prey interactions [4]. The main driving force behind this patterning and complexity involved higher levels of three‐dimensional tissue organisation, specification of cell identity, differentiation and organ patterning [5]. Understandably, due to the comparative ease with which they can be detected, most attention has been focussed on the evolution and diversification of gene‐coding regions in the evolution of metazoans [6]. A good example is the *Hox* genes that have undergone multiple rounds of duplication and clustering during vertebrate evolution thus contributing to the myriad of diverse body plans characteristic of vertebrates [7]. During their embryonic development, even the most basal vertebrates develop extremely complex body forms whose organisation requires the finely coordinated interaction and differentiation of hundreds of different cell types [8]. Since the development of technologies such as *in situ* hybridisation, and most recently, single‐cell RNA‐seq [9], it has been observed that these intricate cellular interactions are accompanied by similarly precise and complex patterns and timing of gene expression at the cellular, tissue and organ levels [10]. Gene targeting in diverse vertebrate models demonstrates that many of these genes are indeed critical to proper embryonic development and that their precise cell‐specific expression is as crucial to their role in development and health as is their amino acid structure [11]. The requirement for precision in gene expression is not merely confined to development, but is also a characteristic of adult homeostasis governing physiologies such as the immune response [12] and brain functions such as appetite [13], reward systems and mood [14]. Unfortunately, compared to our understanding of genes and protein function, slow progress has been made in understanding how the genome controls the cell‐specific expression of these genes as well as their roles in physiology and behaviour. This presents a problem as the vast majority of the tens of thousands of disease‐associated variants discovered in the human genome are found outside of protein coding regions and instead reside within the 98% of the previously dismissed ‘Junk’ genome, that does not encode proteins [15]. This component of the genome is now known to contain most of the information required to direct the cell‐specific expression of genes essential for the development and control of multicellularity.

The current review seeks to critically analyse what is known about the cis‐regulatory elements within the human genome that control the cell‐specific expression of genes essential for health and how they can be detected. We will also use specific examples to explore efforts to ascertain the effects of allelic variation and epigenetic modification on their activity. These regulatory elements will be referred to here as context‐dependent cis‐regulatory elements (cdCREs).

We will start by summarising how the genomes of early single celled eukaryotes have changed from being compact genomes, mainly composed of gene‐coding regions, to the sprawling genomes of vertebrates that are peppered with the thousands of cdCREs required to delimit and control the cell‐specific expression of genes during embryonic development and adult homeostasis. We will also explore the zoo of different cdCREs that are necessary for modulating gene expression and will outline some of the current methods available for their identification. We will examine the case for regarding the regulatory genome as being the largest reservoir of disease‐causing variants in the human genome and, finally, how the regulatory genome acts as a nexus between the genetic and epigenetic influences controlling health and disease susceptibility.

## Bringing order to chaos; multicellularity and the role of cdCREs

It can be argued that evolving the ability of cells to form multicellular communities, and for these cells to communicate, diversify and differentiate to form the huge complexity of metazoan tissues, organs and body plans, was one of the most important steps in human evolution [16]. However, debates continue over the precise nature of the evolutionary processes which allowed the development of this multicellular complexity. Intriguingly, a large number of genes previously thought to be essential for multicellularity, and thereby thought unique to metazoans, such as cell adhesion machinery, developmental transcription factors (TFs) and endocrine cell–cell communication proteins, exist within the genomes of unicellular organisms such as *Capsaspora owczarzaki*, a unicellular relative of metazoans, thus providing a critical point of comparison for understanding the evolution of regulatory complexity [17]. This surprising observation suggests that these genes may have evolved prior to multicellularity and had been co‐opted and amplified through processes including gene duplication events, to contribute to metazoan pattern formation [17]. Despite the unexpected genetic complexity apparent in the *C. owczarzaki* genome, a notable difference between the genomes of unicellular and multicellular organisms lies in the size and structure of their noncoding genomes [18]. For example, a substantial majority of the *C. owczarzaki* genome could be classed as gene‐coding, untranslated region or intronic with only 4% defined as ‘distal intergenic’; an area of the genome populated by regulatory elements in higher vertebrates. This contrasts with the genomes of multicellular organisms where distal intergenic DNA makes up 15% and 58% of the *Drosophila* and human genomes, respectively. Moreover, Sebe‐Pedros *et al*. [18] report that regulatory sites in *Capsaspora* are significantly smaller, lie closer to the 5′ end of genes, bind fewer transcription factors (TFs) and are more uniformly distributed in comparison to *Drosophila* and humans. It was also observed that, although distal regulatory regions, as identified using histone markers (H3K4me1, H3K27ac, etc, to be discussed later), are extremely common in human and *Drosophila* intergenic DNA, they are almost absent in the *C. owczarzaki* genome. These observations suggest that, in addition to amplification of specific sets of gene families, a major driving force in the evolution of the structure, organisation and complexity of multicellular species was the parallel evolution of the regulatory genome.

The importance of the noncoding regulatory genome in gene regulation has been readily apparent to developmental biologists for many years as embryonic development involves the tight choreography of cell division, cell–cell communication, cell migration, morphological transition and differentiation required to produce even the simplest metazoans [19]. Indeed, since the development of recombinant DNA technology, developmental biologists have led the way in identifying the critical roles played by distal regulatory regions in all developmental pathways [20]. The lead that developmental biologists hold in understanding the roles of gene regulation in form and function is aptly demonstrated by studies of embryonic limb bud polarisation [19], bat wing development [21] and stickleback fish spines [22]. However, the role of gene regulation in human health and susceptibility to disease has not been so clear. Thus, recognition of the role of gene regulation in human disease development has only drawn attention relatively recently thanks to the realisation that polymorphisms associated with most complex human diseases do not lie in the coding regions of genes, as originally hoped. Instead, these disease‐associated SNPs lie in the noncoding intronic and intergenic components of the genome where the hundreds of thousands of enigmatic cdCREs lurk and whose function is poorly understood and will be discussed in the next section [15].

## The GWAS dilemma; evidence for the role of the noncoding genome in health and disease

The sequencing of the human genome early in the new millennium triggered an extraordinary wave of advances in the use of gene chip technologies to analyse hundreds of thousands of allelic variants in huge patient cohorts, often numbering hundreds of thousands or millions of individuals [23]. These analyses have included genome‐wide association studies (GWAS) that have greatly altered our understanding of the genetic architecture of heritable human characteristics including disease susceptibility [24]. Indeed, GWAS has facilitated the identification of thousands of different allelic variations with strong associations (*P* < 1 × 10^−6^) to specific disease phenotypes. GWAS has generated several notable successes, such as identifying a crucial role of the IL‐12/IL‐23 pathway in Crohn's disease, that has enabled the development of more effective therapies [25]. Yet, the majority of significant GWAS associations highlight genes whose roles in disease progression are often difficult to reconcile with a given disease. For example, despite the serotoninergic system being one of the main treatment targets for depressive disorders, large‐scale meta‐analyses of GWAS have struggled to identify strong associations with depression and genome regions encoding components of the serotoninergic system [26]. There may be many reasons for this observation. The first is that even the most highly statistically powered GWAS currently lack the resolution to pinpoint causative loci because they are mostly based on gene chip technologies that cannot include all loci in the human genome. Instead, it is widely held that GWASs serve to identify ‘marker’ SNPs within regions of the genome in strong linkage disequilibrium (LD, where clusters of alleles consistently cosegregate through the population; also known as LD blocks) which can often cover tens or hundreds of kilobases and can include thousands of other SNPs; any of which may be the actual causative loci [27]. Although challenges will be met with analysing the huge amount of data that will be generated, it is hoped that the gradual replacement of GWAS with whole genome sequencing (WGS) of patient DNA will enable greater resolution for the identification of causative variants [28].

Another contributing factor which may reduce the apparent resolution of GWAS is that, unlike classical Mendelian inheritance (single loci affecting specific phenotypes), many complex diseases are not caused by one or two allelic variants but manifest through the combined input of many tens or even hundreds of different loci [15]. This has been called the ‘infinitesimal model’ where, hypothetically, the majority of SNPs in the genome may contribute to a trait or disorder to some degree [15]. Moreover, the combined contribution of statistically significant GWAS ‘hits’ seldom account for the observed genetic variance. For example, it was shown that the 697 genetic loci, that were significantly associated with height, only accounted for 16% of the phenotypic variance [29]. Instead, it is becoming apparent that the disease ‘load’ is shared by tens or hundreds of loci, each of which only contribute by a small amount [15, 30]. Thus, the relative penetrance of various disease states may vary within the population depending on the genetic ‘card hand’ of disease‐contributing alleles ‘dealt’ to each individual in the genetic ‘poker game’. Nonetheless, the results of thousands of GWA studies have suggested that the human genome contains an important source of information that does not encode proteins but is critical for development and health [15, 30]. We will now examine a critical, but relatively poorly understood, components of the noncoding genome; context‐dependent cis‐regulatory elements.

## Enigmatic control freaks; what are CREs and how do they work?

The term ‘cis‐regulatory element’ is an umbrella term that covers several different functional sequences and includes promoters, enhancers, silencers and insulators. Because of their proximity to transcriptional start sites (TSS), promoters are the best understood regulatory regions in the human genome. They mostly comprise short (~ 100 bp) sequences of DNA next to the TSS of genes needed to bind RNA polymerase II and the transcriptional pre‐initiation complex [31]. Critically, their functions are orientation‐ and distance‐dependent on the TSS that they control. Promoter sequences also comprise a smaller proportion of the human genome than coding regions and are often unable to support tissue specificity on their own [32]. Promoters therefore require the presence and activity of other sequences such as enhancers and silencers to maintain the levels of gene expression appropriate for development and health [33]. Enhancers and silencers are functionally distinct from promoters in that they are distance‐ and orientation‐independent with respect to TSSs. Nevertheless, despite the real possibility that they represent the largest reservoir of information in the genome, there is still considerable confusion about how enhancers and silencers can be reliably identified, their significance for development, health and disease and how their activity is affected by polymorphisms or the environment. They also likely to comprise the majority of the regulatory elements covered by the term cdCRE.

**Fig. 1:**
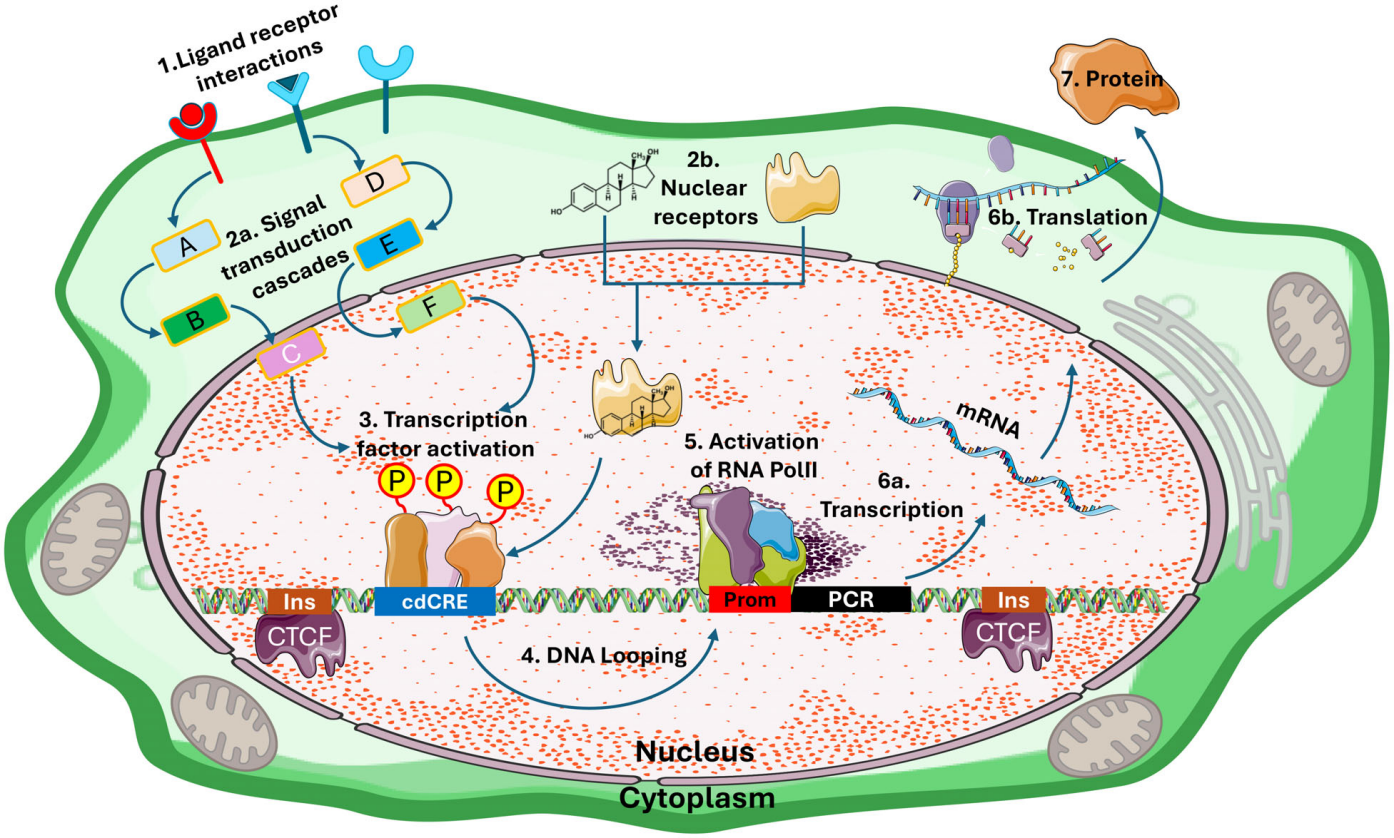
AQ5CdCREs are essential components that permit the genome to interpret the flow of information between, and within, cells. A simplified diagram illustrating the flow of information within cells of multicellular organisms. Step 1. Cell surface receptors are bound by ligands secreted from other cells. This stimulates the activation of signal transduction cascades (step 2a) whereby cytoplasmic proteins (A–F) are post‐translationally modified or where nuclear receptors bind their ligands (step 2b). Step 3 involves the post‐translational modification (represented by yellow Ps) of transcription factor proteins (TF) by activated signal transduction proteins thereby stimulating their binding to cdCREs (Blue). Step 4. Once bound to cdCREs, activated TFs are brought into the proximity of RNA polymerase II (RNA polII) bound at promoters (red) by looping of intervening DNA (see Fig. 2 for mechanism). This stimulates RNApolII activity (step 5) thereby driving gene expression from protein coding regions (PCR, Black) that consists of transcription (step 6a to produce mRNA) and translation (step 6b to produce proteins step 7). Insulator sequences (Ins) bound by CTCF protein have been included to demonstrate their spatial relationships to cdCREs such as enhancers and promoters.

Collectively, cdCREs are essential components of the genomes of multicellular organisms as they allow the genome to interpret the flow of information between and within cells (Fig. 1). This flow of information is initiated at the cell surface by the binding of ligands, including hormones and other signalling peptides, to cell surface receptors. Activated receptors at the cell surface initiate signal transduction cascades that result in the post‐translational modification of DNA binding TFs proteins. Activated TFs then bind CREs within the genome, such as enhancers or silencers, thereby activating, or repressing, their context‐specific activity (Fig. 1). Exceptions to this are nuclear receptors, such as the oestrogen, androgen and corticosteroid receptors, which act as transcription factors when bound by their specific ligands [34] (Fig. 1).

It is worth considering that the interaction and binding of TFs to CREs is an essential component of the normal function of CREs in development and health. Indeed, in view of the observation that > 90% of GWAS‐associated SNPs occur within the noncoding genome, alterations in TF‐CRE binding, due to allelic differences, may represent the major driving force in heritable disease susceptibility within human populations [35]. Many technologies have been developed to characterise the binding of TFs to DNA and to assess the effects of allelic variants on these interactions. One of the earliest was the electrophoretic mobility shift assay (EMSA) that could resolve the interactions of TFs and DNA on polyacrylamide gels [36]. This method involved the radiolabelling of target DNA which was exposed to either purified protein or cell nuclear extract. DNA‐protein samples were then loaded onto polyacrylamide electrophoresis gels whereby any protein that bound the radiolabelled DNA ‘retarded’ its migration through the gel matrix (EMSAs are also known as ‘gel retardation assays’ or ‘gel shift assays’) [35]. Another more current and more widely used technique is the chromatin immunoprecipitation (ChIP) assay [37] which relies on the use of an antibody raised against a specific DNA binding protein which can be immobilised onto a solid matrix [38]. Chromatin for analysis is crosslinked with formaldehyde, to ‘freeze frame’ TF‐DNA interactions, then sheared into small fragments by sonication. These DNA‐protein fragments are then incubated with specific immobilised antibodies, washed, eluted and analysed using quantitative polymerase chain reaction (QPCR) analysis. Alleles which reduce TF affinity will result in a lower recovery of DNA and a lower QPCR signal. Many excellent examples have been published that include the use of these technologies to determine the effects of disease‐associated allelic variation on TF‐DNA interactions [39, 40, 41].

One of the most intriguing, but troubling, aspects of cdCRE biology is that enhancers and silencers can influence promoter function from enormous distances within the genome. For example, one enhancer that controls the expression of the *Sonic Hedgehog* gene (*SHH*) in the developing limb bud was found to lie over one megabase (Mb) away from the *SHH* gene and, disturbingly for those trying to make sense of genome structure and function, within the intron of another gene (*LMBR*) whose expression is not affected by the enhancer [19, 42]. Indeed, it is now recognised that the distance that cdCREs lie from the genes that they control may be a major evolutionary force restraining the fragmentation of synteny blocks between often widely diverged species [43, 44].

**Fig. 2:**
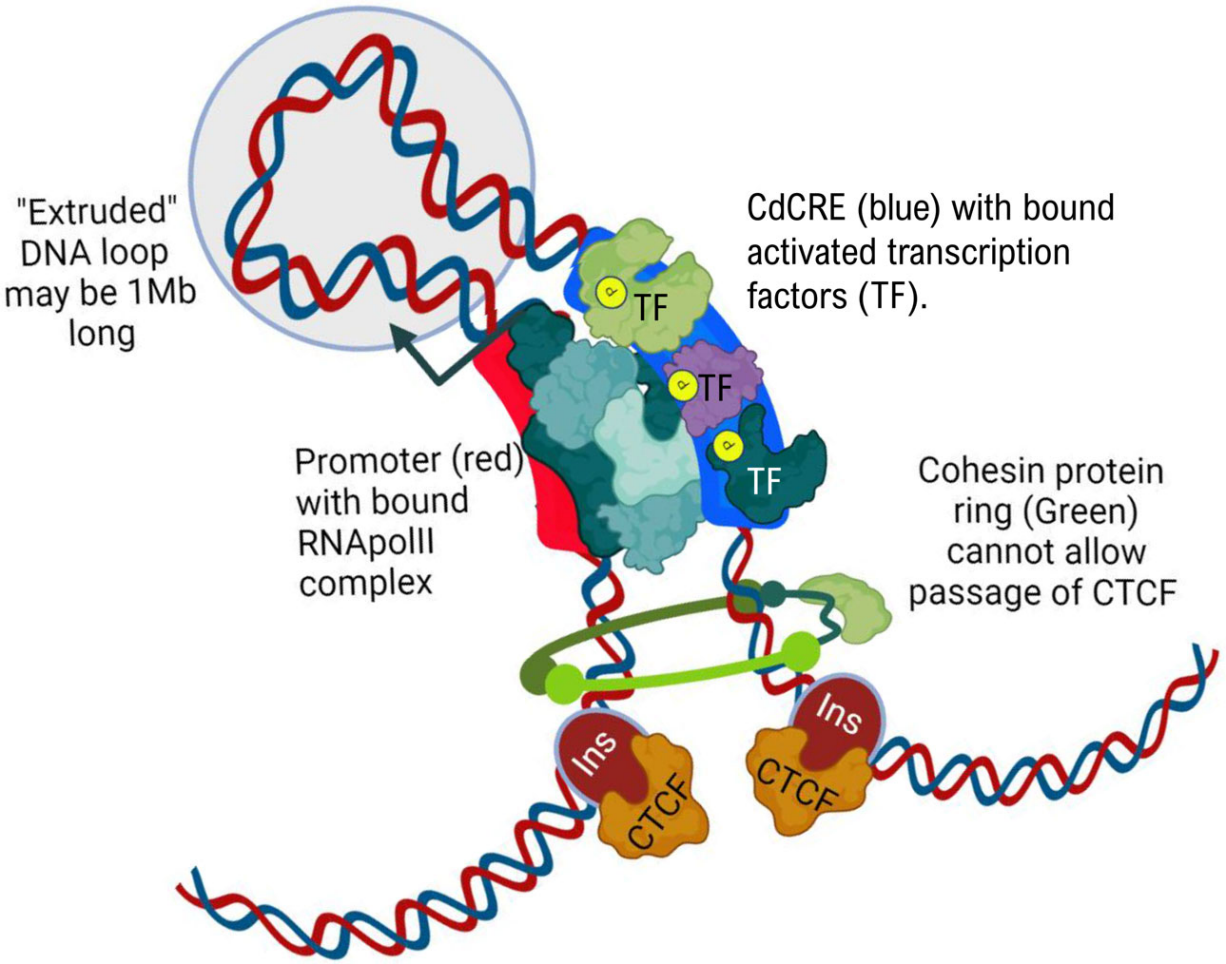
Remote cdCRE sequences interact with promoter sequences by a process of loop extrusion through cohesion protein rings. Loops of DNA are fed through a cohesin protein ring (represented in green) and stop once CTCF (bound at insulator sequences (Ins)) are encountered. This process is thought to bring proteins bound at promoters (red) and cdCREs (blue) into close proximity, stimulating RNApolII activity and transcription of mRNA in specific cells and at specific times.

How do cdCREs overcome these distances to modulate promoter activity? The loop extrusion model is currently the most widely accepted hypothesis (Fig. 2). As the name suggests, this model involves the extrusion of loops of DNA through a circular protein complex called cohesin that was initially discovered due to its role in sister chromatid pairing during meiosis [45]. The extent of this loop extrusion in modulating transcription is determined by the interaction of CCCTC‐binding factor (CTCF) proteins with insulator sequences (also known as TAD boundary sequences) which, together, delimit regions of the genome known as topological associating domains (TADs) (Fig. 2) [46]. TADs were discovered using Hi‐C which is a technique based on chromatin conformation capture. Briefly, this technique involves the formaldehyde cross‐linking of chromatin in cultured cell lines or cells from dissociated primary tissues followed by a fragmentation and a re‐ligation step [47]. Sequencing of these ligated products provides a ‘snapshot’ of long‐distance interactions within the genome. For example, the *SHH* gene and its limb enhancer are precisely encompassed within a TAD whose structure is conserved in both humans and mice [48].

## Revisiting a perceived dogma; Identifying enhancers and silencers using the histone code

Identifying and characterising the functional characteristics of CREs has become one of the most difficult tasks in biology as, in contrast to the three‐letter codon usage of the protein‐coding genome, predicting the functional components of the noncoding regulatory genome has been very challenging. Most efforts to identify and characterise functional components within the noncoding genome, as typified by the ENCODE consortium data release in 2012 and many subsequent studies, have relied on identifying these components based on a ‘Histone code’ [49]. For example, accepted markers of active enhancers include changes in the methylation and acetylation status of specific lysine residues (lysines 4 and 27) within histone H3, (abbreviated to H3K4me1 and H3K27ac) [50]. These modifications can be detected using ChIP as described above. In addition, the development of next‐generation sequencing (NGS) technologies has allowed for the sequencing of all DNA–protein complexes bound by each immobilised antibody thus providing genome‐wide snapshots of transcription factor binding and histone methylation status. Using ChIP‐seq (ChIP analysis involving NGS) approaches, the ENCODE consortium has been able to construct detailed maps of active functional elements such as promoters (H3K4me3), enhancers (H3K4me1, H3K27ac and P300), silencers (H3K27me3) and insulators (CTCF binding) in a wide variety of different cell lines, cancers and cell types [51]. These observations were supported by techniques designed to identify open chromatin; DNA regions with reduced histone interaction, another diagnostic marker of transcriptional activity within the genome (DNase‐seq, ATAC‐seq) [49, 52, 53] and concluded that up to 80% of the human genome had function. Furthermore, based on these studies, alignment of multiple higher vertebrate genomes concluded that only 5% of the enhancers and silencers identified were conserved through evolution [49]; this conclusion was further supported by other laboratories using H3K27ac analysis of primary hepatocyte chromatin and is consistent with a hypothesis that enhancers evolved and changed rapidly during vertebrate evolution and speciation [54]. Encouraged by the ENCODE data release and the plummeting cost of NGS‐based technologies, the field of functional genomics has generated many terabytes of data that claims to further map functionality within the human genome using histone marks in transformed cell lines [55]. Furthermore, many attempts have been made to use this data to interrogate the similarly huge and rapidly growing GWAS data sets being generated by clinical geneticists [56].

In an effort to make sense of this accelerating ‘deluge’ of data, several attempts have been made to develop ‘high throughput’ technologies, based on either ‘machine learning’ [57] or cell culture paradigms [58, 59]. Although elegant in their design, these efforts have yet to successfully bridge the gap between the discovery of new regulatory elements and our understanding of what role these CREs play in health and disease. One of the main issues that ‘high throughput’ or ‘massively parallel’ approaches face is the high likelihood that many CREs may not be active within the cell lines used to identify and characterise them because of the high‐context dependency of their activity. Even more disturbing is the possibility that false signals may be generated in regions of the genome without regulatory functions within the greatly mutated, transformed and immortalised cell lines used. For example, a functional study of ENCODE‐predicted enhancers showed that only 26% of these ‘enhancers’ were active [60]. In addition, deleting the genes encoding the Trr proteins responsible for adding enhancer‐specific H3K4me1 markers from the Drosophila genome resulted in normal development thus questioning the requirement for this marker in enhancer activity [61].

The tendency to merely define CREs as being landing sites for specific histone markers, as a proxy for functional elements, must be challenged if we are to progress our understanding of CRE biology in health and disease. In addition, extrapolating major conclusions about CRE activity based on a small number of chromatin markers may not be that helpful in understanding their role [62]. Consequently, it has been suggested that defining an enhancer based only on ‘enhancer‐specific’ or ‘promoter‐specific’ chromatin modifications, or other markers such as p300, should only be accepted if supported by *in vivo* functional data [63]. The next section will re‐examine what CREs are from a more functional perspective and challenge evidence previously used to suggest their lack of evolutionary conservation.

## If it isn't broken, don't fix it; re‐evaluating the case for conservation

The dogma surrounding CREs such as enhancers and silencers, that originated in the 1980s, stated that they were sequences required to upregulate or downregulate the activity of gene promoter regions [33, 64]. Within an individual cell or a homogeneous clonal culture of transformed cells using viral CREs, this may indeed be the case. However, within a highly complex and differentiated multicellular environment, these sequences play another important role that is often overlooked, that of modulating the context‐dependent expression of the genes required to pattern the complex body plans of metazoans and to keep them alive and healthy [65]. Unfortunately, attempting to identify active enhancers using biochemical histone marks within transformed or immortalised cell cultures may not represent the most appropriate context and has only had very limited success in identifying cdCREs with demonstrable *in vivo* function [60]. With this in mind, there is a case for revisiting evolutionary conservation as a method for identifying functional cdCREs.

Several layers of evidence point to the possible evolutionary conservation of cdCREs within higher vertebrates. The first relates to the commonality of body patterning and physiologies and behaviours, such as appetite, the inflammatory response and fear/anxiety, that are shared by all higher vertebrates and rely on the same cells and tissues. Moreover, many of the organ systems and tissues that regulate these behaviours/physiologies express many of the same genes in the same cell types [66, 67]. The conservation of the spatial gene expression domains evident in most higher vertebrates suggests that the genomic processes and regulatory sequences that control the expression of these genes may have also been conserved. This is supported by previous high‐throughput studies of conserved candidate enhancer sequences analysed using transgenic mouse reporter lines which demonstrated that > 70% of noncoding regions, conserved between mouse and human, had clear enhancer function in specific tissues [68]. Other examples of highly conserved enhancers include those that coordinate expression of the interleukin genes [69], the *SHH* gene [19], the *pro‐opiomelanocortin* gene [70], Pierre‐Robin‐sequence PRS‐enhancer [71], the *Hox* gene clusters [72] and the *LMO1* gene [41]. Consistent with these observations, more recent ENCODE studies, that have moved away from single‐cell‐based analyses to analyse primary cells derived from model organisms, have reported a much higher level of enhancer conservation; up to 65% as opposed to the 5% previously reported [73]. Intriguingly, they further concluded that the most conserved regulatory elements were the most important for gene regulation and that newly evolved elements only contributed weakly [73].

Whilst there is a growing consensus that combining the histone code and open chromatin with comparative genomics might provide a future direction for identifying cdCREs, there is little doubt that characterising their biology and their roles in health and disease using transformed cells or even primary cell monoculture alone is less than adequate. The next section will set out a case for exploring the function of conserved cdCREs using more relevant multicellular models.

## An inconvenient truth; the need to study cdCREs in a multicellular context

Whilst many of the cdCREs that control our development, health and susceptibility to disease have been conserved throughout vertebrate evolution and can be identified as a result, choosing an appropriate method for their subsequent analysis is still widely debated. Clearly, cell culture strategies alone are far from adequate for examining the roles of cdCREs in development, health, behaviour and disease. Other forms of cell culture such as the formation and analysis of organoid cultures, that claim to reproduce three‐dimensional organ‐like ‘organoids’ following chemical stimulation of stem cells, hold promise in understanding developmental processes [74]. However, organoids are unable to determine the role of cdCREs in the majority of physiologies or behaviours common to higher vertebrates and which contribute to the disease process in humans.

**Fig. 3:**
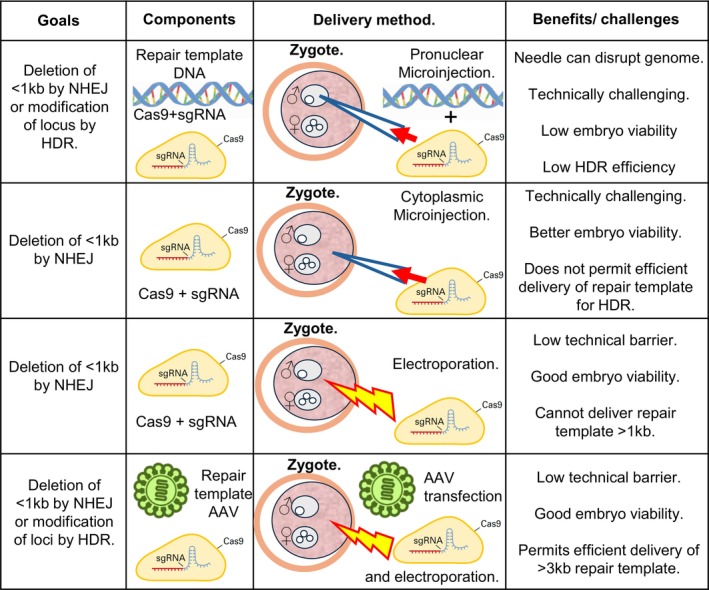
Cas9/CRISPR manipulation of the mouse genome is an efficient and effective method of understanding the roles of cdCREs in higher vertebrate physiology and behaviour. An illustration of the developments and refinements in the use of CRISPR/CAS9 in the manipulation of cdCREs in mice over the past 10 years, the goals of each protocol, the components used, zygote genome delivery methods and benefits and challenges of each protocol. AAV, adeno‐associated virus (green); HDR, homology‐directed repair; NHEJ, Nonhomologous end joining; sgRNA, single guide RNA.

An alternative approach is the manipulation of the genomes of animal models such as mice. Genetically manipulated animal models have been used for over 40 years to explore the cell‐specific activity or context dependency of cdCREs using microinjection of LacZ or GFP expressing reporter plasmids into the pronucleus of one‐cell mouse embryos [68, 75, 76, 77]. More recently, the development of CRISPR‐Cas9 technology has revolutionised our ability to rapidly delete or manipulate identified cdCREs to determine their roles in development, physiology and behaviour [78]. The CRISPR‐Cas9 system takes advantage of a bacterial (*Streptococcus pyogenes*) protein called Cas9 that can be instructed to cut DNA molecules at specific sequences *in vivo*. These instructions are given to Cas9 in the form of a single guide RNA (sgRNA) whose sequence can be changed to hybridise with the target sequence (Fig. 3). sgRNAs form a ribonucleoprotein (RNP) complex with Cas9 that has been engineered with a nuclear localisation signal that targets the protein‐RNA complex to the cell nucleus. Once in the nucleus, the Cas9‐sgRNA identifies and cuts the target sequence specified by the sgRNA [79] (Fig. 3). The only factor limiting sequence choice is the need for the target sequence to be next to a protospacer adjacent motif (PAM) sequence, which, in the case of Cas9 derived from *S. pyogenes* is NGG [79]. It is important to understand that the CRISPR system only acts as a programmable molecular scissor and that the host cell must carry out the repair. The DNA repair pathway most often recruited by cells to repair broken DNA is the nonhomologous end joining (NHEJ) pathway which is not accurate and can remove or insert inappropriate DNA sequences during its repair [80] (Fig. 3). A more accurate pathway used by the cell is the homology‐directed repair (HDR) route which can be harnessed if repair template DNA, containing regions of DNA homologous to the target sequence, is introduced into the cell at the same time as the Cas9 RNP complex [81] (Fig. 3). Whilst pronuclear microinjection of CRISPR components into the pronucleus has been the most widely used method of delivery, valid concerns have arisen around the genomic damage that insertion of a glass needle into the nucleus may incur (Fig. 3). An alternative approach involves injection of RNP complexes into the zygote cytoplasm (Fig. 3) which has also led to success but is still very labour intensive [82]. More recently, considerable progress has been reported using electroporation of RNP complexes into embryos which is much less labour intensive (Fig. 3) and has the added advantage of preventing genome damage associated with needle insertion into the pronucleus [83]. Moreover, previous problems encountered with the electroporation of large DNA repair templates have been circumvented with pre‐infection of zygotes with repair template DNA contained within an adeno‐associated viral (AAV6) package [84] (Fig. 3). This method of repair template delivery is extremely efficient and circumvents issues associated with pronuclear microinjection such as DNA shearing and reduced embryo viability. Indeed, our recent attempts at using this technique, in combination with electroporation of CAS9‐sgRNA RNP complex has allowed the generation of CRE humanised mice with a 50% efficiency. The streamlining of GA mouse production using electroporation of RNP components combined with AAV6 transfection of repair templates has the potential of revolutionising our understanding of conserved cdCREs *in vivo* and represents a critical step in understanding their role in health and disease.

Clearly, the advantages afforded by deleting putative cdCREs in animal models, and AAV‐CRISPR driven humanisation, can only be realised if the cdCREs in question are common to both humans and the model animal to be used. For example, it will be very difficult, if not impossible, to model the involvement of a cdCREs element detected using histone marks or open chromatin in transformed human cell lines if an equivalent cdCRE does not exist in other species. Indeed, a major advantage of focusing on putative conserved cdCREs is that the activity and roles of these conserved enhancers can be explored experimentally *in vivo* in a way that nonconserved enhancers cannot. Whilst there are likely to be regulatory sequences unique to humans, it is also critical that we gain a strong grasp of the regulatory mechanisms controlling gene expression common to all higher vertebrates to provide a platform on which to build our understanding of human‐specific gene regulation. Thus, thanks to the comparative ease of manipulating the genomes of model organisms such as mice with the CRISPR‐Cas9 genome editing system, there has never been a better time to study the role of conserved human cdCREs in placental mammals.

## Cases in point; cdCREs, neuropeptides and behaviour

**Fig. 4:**
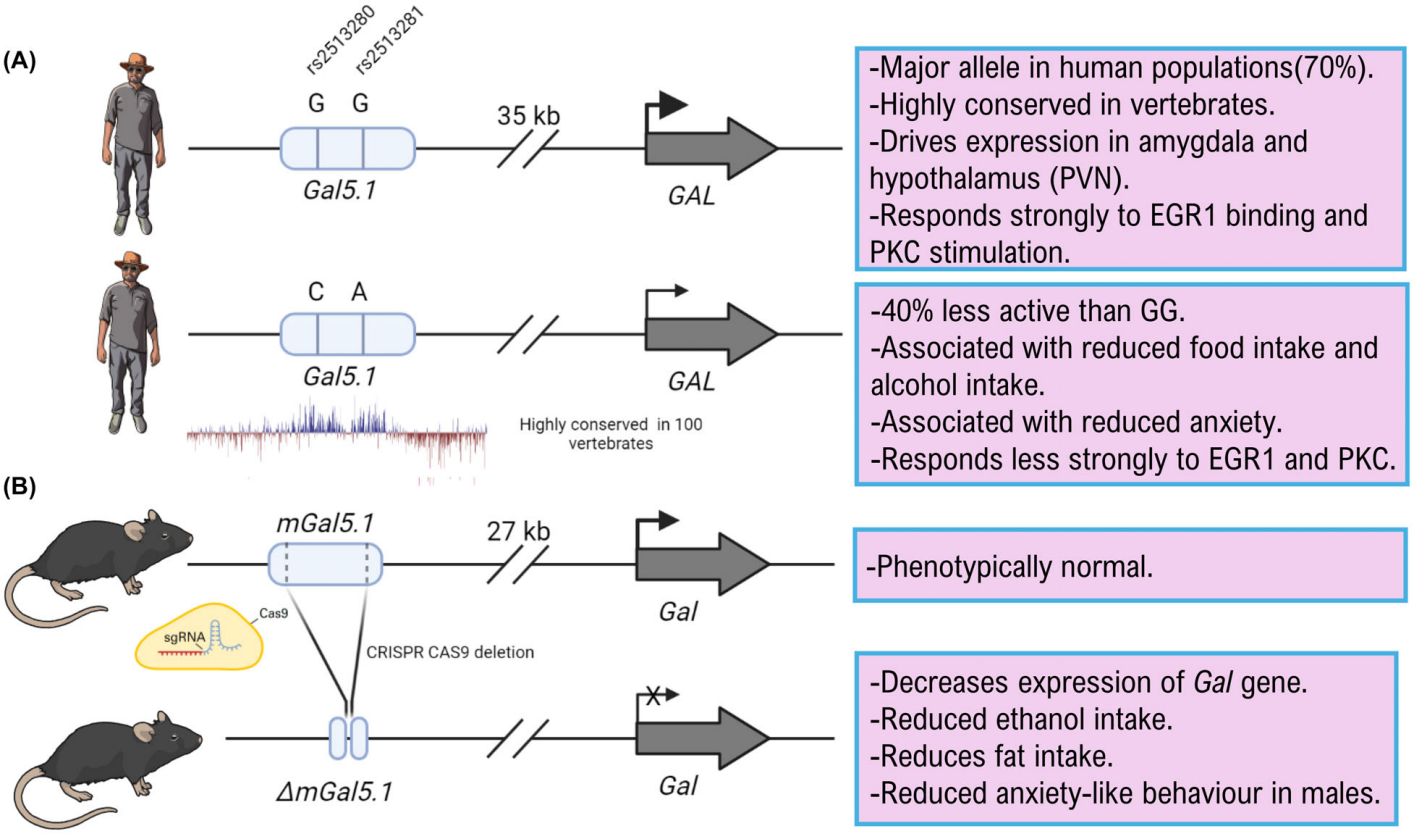
The highly conserved and polymorphic GAL5.1 enhancer modulates ethanol intake and anxiety in mice and humans. Analysis of the GAL5.1 enhancer in human populations (A) and in mice (B). (A) Demonstrates allelic frequency of each allele in the population (GG and CA) and the effects of each allele on enhancer activity. It includes information on the association of each allele to changes in consumptive behaviour and mood. A graph demonstrating levels of conservation in 100 different vertebrate species is also shown where the *X*‐axis reflects genomic distance (2 kb) and the *Y*‐axis reveals levels of conservation between 50% and 100%. (B) Analysis of the mGal5.1 enhancer in mice demonstrating the role of the enhancer in wild‐type animals and the effects of CRISPR‐CAS9 deletion on the expression of *Gal*, as well as consumptive behaviours and mood.

To test the hypothesis that conserved enhancers modulate physiologies and behaviours important in vertebrate health such as fat intake, ethanol intake and anxiety we examined the regulation of the *GAL* gene, which encodes a neuropeptide (galanin) expressed in the hypothalamus and amygdala and is known to modulate these behaviours [85, 86]. Previous association studies in humans found SNPs around *GAL* associated with excess ethanol intake and anxiety in men [87]. To identify the regulatory mechanisms controlling this gene we used comparative genomics to find a highly conserved polymorphic region of DNA, called GAL5.1, that had been conserved in all higher vertebrates and lay 43 kb from the *GAL* TSS (Fig. 4A). Generation of reporter mouse lines and analysis in primary cell lines demonstrated that GAL5.1 supported reporter expression in the same cells that express galanin in hypothalamus and amygdala and was functionally altered by allelic variants [88]. Intriguingly, analysis of the UK biobank further suggested that allelic variations within GAL5.1 were associated with alcohol abuse and anxiety in men [89]. Using CRISPR removal of the GAL5.1 cdCRE, and behavioural analysis in mice, we were able to show that GAL5.1 also controlled alcohol intake and anxiety in male mice, an observation reflecting what we had seen following interrogation of the UK Biobank (Fig. 4B) [89]. A critical finding of our study was that GAL5.1 bound the EGR1 transcription factor and could be activated by the protein kinase C signal transduction pathway in an allele‐specific manner (Fig. 4). These studies strongly support the hypothesis that cdCREs required to support context‐specific gene regulation can be highly conserved [19, 41, 68, 69, 71]. It is also highly likely that this conservation reflects the specific order of transcription factor binding site syntax [90] that is required to support the sophisticated expression patterns required for normal development and health. Indeed such complex TF binding syntax is extremely unlikely to evolve spontaneously as previous studies cell‐based studies may have suggested [49, 54].

## Climbing mount GWAS; translating GWAS data into a functional understanding of noncoding variants and disease

**Fig. 5:**
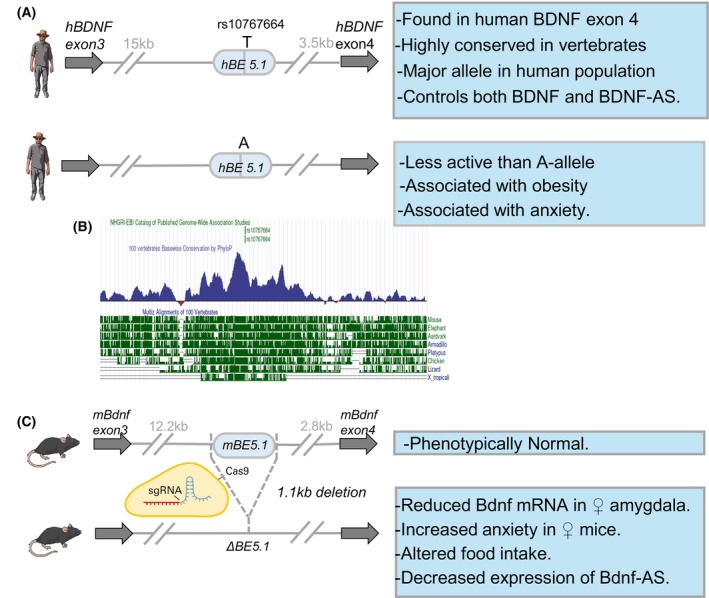
An ancient polymorphic regulatory region within the *BDNF* gene associated with obesity modulates anxiety‐related behaviour in mice and humans. Analysis of the BE5.1 enhancer in human populations (A) its conservation in vertebrates (B) and its role in mice (C). (A) demonstrates the different alleles of BE5.1 present in the human population, the distance that BE5.1 lies in relation to exons of the *BDNF* gene and the known associations of allelic variants of BE5.1 with obesity and anxiety. (B) A graph from the UCSC browser demonstrating levels of conservation of BE5.1 in 9 highly divergent vertebrate species compared to human DNA. Light green numbers and bars refer to GWAS‐associated SNPs. Blue peaks represent relative levels of conservation, and dark green bars next to species names reflect depth of conservation back to amphibians (Xenopus tropicalis, diverged from humans 350 million years ago). (C) The results of a CRISPR deletion of the BE5.1 enhancer in mice demonstrating its role in regulating sex‐ and cell‐specific gene expression levels food intake and anxiety‐related behaviours.

A critical step in developing our comprehension of how genetic variation within the human population alters susceptibility to disease will involve translating the ever‐growing mountain of GWAS‐associated noncoding SNPs, that represent > 95% of all GWAS SNPs, into a functional appreciation of the noncoding genome in health and disease. In an effort to identify the roles of noncoding GWAS‐associated SNPs in disease, we explored the role of GWAS‐identified SNPs lying within and around the gene encoding the brain‐derived neurotrophic factor (*BDNF*). BDNF is a 118 amino acid peptide expressed in the hippocampus, hypothalamus and amygdala where it plays a critical role in modulating behaviours such as food intake and anxiety [91, 92, 93, 94, 95, 96, 97]. BDNF does so through its modulation of neuronal survival, proliferation and differentiation of neurons and glial cells as well as axon growth, synapse formation, transmission and plasticity which it mediates through the Tropomyocin Receptor Kinase B (TRKB) and neurotrophin factor (p75NTR) receptors [98]. Multiple GWAS studies have identified a large number of polymorphisms, in and around the *BDNF* locus, that have been associated with obesity [91, 92, 93, 94], anxiety [95, 96, 97] and addictive disorders [99, 100]. However, only one of these polymorphisms (rs6565) affects the coding region of *BDNF* [100] suggesting that most of the contribution of *BDNF*‐associated SNPs in disease may not involve changes in its primary amino acid sequence. In order to understand the effects of these polymorphisms on disease susceptibility, we focused on a SNP strongly associated with obesity, that occurs in a highly conserved putative cdCRE (BE5.1), (Fig. 5) [101]. CRISPR deletion of BE5.1 significantly altered food intake suggesting a role in modulating appetite [102] (Fig. 5). Furthermore, female BE5.1 KO mice expressed higher levels of *BDNF* mRNA in the amygdala and were significantly more anxious, a behaviour reversed using diazepam [102] (Fig. 5). Intriguingly, analysis of expression quantitative trait loci (eQTL) in humans (GTEx database) showed that allelic variants of rs10767664 altered expression and splicing of *BDNF*‐antisense (BDNF‐AS) transcripts [102]. In parallel, we showed that the BDNF‐AS in mice was significantly decreased by BE5.1 deletion suggesting a novel mechanism for *BDNF* gene regulation in anxiety involving antisense RNA [102] (Fig. 5). We also found that the A and T variants of BE5.1 drove different levels of activity of *BDNF* promoter IV (BP4) and that analysis of the human UK Biobank cohort demonstrated a greatly increased association with allelic variants of rs10767664 and anxiety [102] (Fig. 5). These experiments further demonstrate that combining comparative genomics with CRISPR‐based *in vivo* analysis represents a robust methodology for identifying and characterising cdCREs that host GWAS‐associated SNPs and which play a role in regulating behaviours including appetite and anxiety.

## The 5th and 6th bases in health and disease; DNA methylation and cdCREs

In addition to DNA polymorphisms within cdCREs, the activity of these elements is also subject to environmentally influenced epigenetic modification. One type of epigenetic modification, DNA methylation of genomes at CpG dinucleotides (5mC), has been widely studied thanks to its changing distribution in the genome in health as well as in response to diseases as diverse as cancer [103], inflammatory diseases [104] and mental health [105]. Most of these studies have focused on gene‐coding regions and CpG islands; genomic regions rich in CpG dinucleotides that lie close to, or within, many promoter regions and which are generally protected from 5mC [106]. Indeed, it has been posited that a major contributing factor in the emergence of higher disease risk in the aging population is gene misregulation as a result of increased methylation of these CpG islands in later life [107]. Although promoter and CpG island methylation are known to repress transcription [108, 109], the effects on cdCRE activity are less clear and the literature is inconsistent with regard to their effects on cdCREs and urgently needs clarification [110, 111, 112]. For example, using DNAseI hypersensitivity, the ENCODE consortium observed a decrease in chromatin accessibility in only 20% of accessible sites as a result of CpG methylation [113]. Furthermore, an analysis of the effects of 5mC on the binding affinity of transcription factors found that only 23% of these experienced reduced binding. By contrast, the binding of 34% of TFs was actually increased by 5mC [114]. The role of DNA methylation in influencing cdCRE activity is also altered by intermediate oxidation products of 5mC that include 5‐hydroxymethylcytosine (5hmC), the product of the Ten‐Eleven Translocation (TET) dioxygenase enzymes [115]. Thus, TET protein‐driven 5hmC deposition is associated with increased transcriptional activity [116] and 5hmC sites are attractive to TFs that activate transcription [117]. Moreover, 5hmC has been associated with active enhancers in stem cells [118, 119, 120] and TET2 deletion from mouse stem cells reduces both 5hmC levels and enhancer activity [119]. Taken together, these studies suggest that the effects of DNA‐methylatin on genome function are far from simple and it is critical that we understand the effects of 5mC and its various degradation products on the activity of the functional components of the genome.

In an effort to understand the influence of DNA methylation on enhancer activity, we explored how early environmental changes such adversity or diet can induce long‐term changes in gene expression. We showed that early life stress (ELS) in rats using short 2 h periods of maternal separation for the first 10 days of life led to a permanent increase in the activity of the *Arginine Vasopressin* (*Avp*) gene expression and peptide in neurons of the paraventricular nucleus (PVN) of the hypothalamus. This increased AVP‐induced stress behaviour in the rats and a specific Avp receptor antagonist abolished this demonstrating this behaviour was driven through Avp. Importantly, the expression of *Avp* in other Avp‐expressing neurons in a neighbouring brain region, the Supraoptic Nucleus (SON) involved in osmolality, were not changed—ELS did not alter serum osmolality, attesting the important cell—and context‐specific regulation of this gene following ELS [121]. Sequence analysis by Harold Gainer of the *Avp* locus in rats, mice and humans, identified conserved cdCREs downstream of the gene, shown to essential for the cell‐specific expression [122]. When measuring DNA methylation across the AVP locus in ELS‐exposed rats, we found decreases in methylation at specific CpG sites within one of these cdCREs together with active histone marks only in the PVN of stressed animals and not the SON [121]. In order to understand how these specific marks were established at this cdCRE, we developed an embryonic stem cell‐derived model of hypothalamic‐like differentiation that we used together with *in vivo* experiments. At the Avp cdCRE in early hypothalamus development, we found binding of polycomb proteins together with Tet proteins and 5hmC that during differentiation were replaced with DNA methyltransferase (DNMT) leading to 5mC at the cdCRE. Levels of Methyl‐CpG‐binding protein (Mecp2) then increased within the PVN around the perinatal period and bound to 5mC within the *Avp* cdCRE in turn recruiting histone deacetylases (HDACs) and further DNMT, indelibly setting in motion life‐long *Avp* activity [123]. Coming back to ELS, we found that around the perinatal period, ELS‐dependent PVN‐specific neuronal activity inhibits Mecp2 function, selectively preventing its binding to the *Avp* cdCRE in PVN and thus ensuring perpetually increased levels of *Avp* activity [124].

**Fig. 6:**
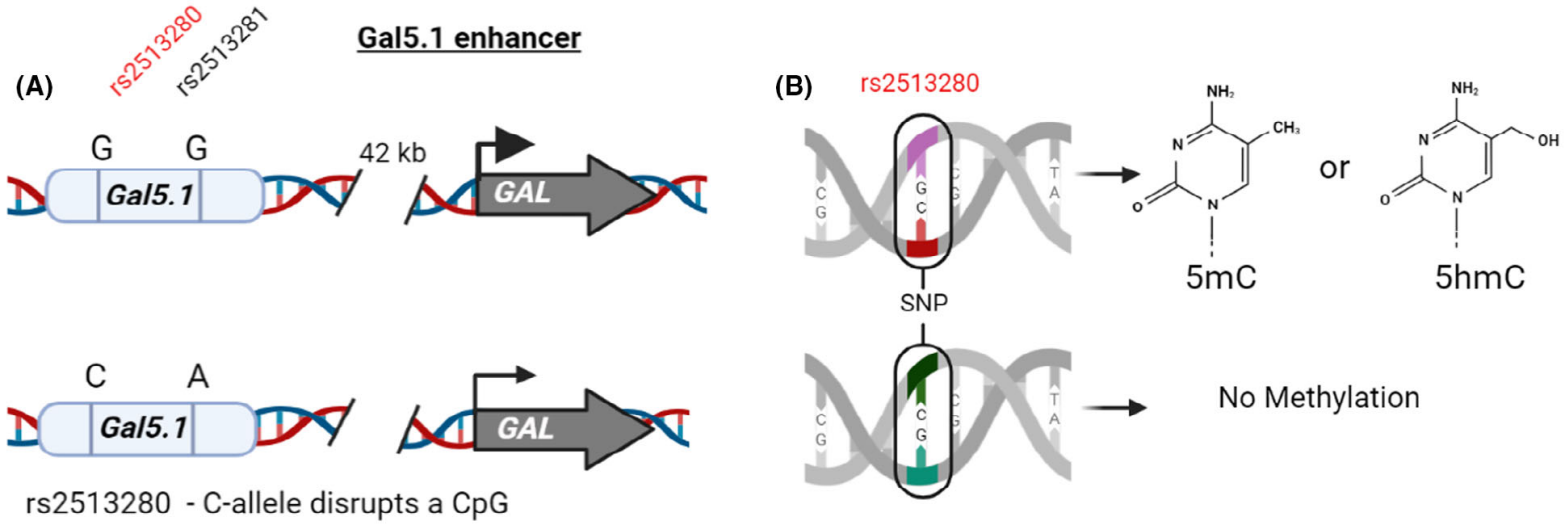
cdCREs can act as a functional nexus between hardwired genetics and environmentally controlled epigenetics. A representation of the human GAL5.1 enhancer showing (A) both of the genotypes (GG or CA) in the human population. (B) The C‐allele of rs2513280 disrupts a CpG changing it to a CpC thereby rendering the enhancer less susceptible to the effects of methylation (5mC) or hydroxymethylation (5hmC).

In addition, we were able to demonstrate that increased fat intake during pregnancy had a significant effect on methylation of the GAL5.1 enhancer in the hypothalamus and amygdala; an interesting observation considering its role in fat intake, ethanol consumption and mood [89]. A notable finding was that different regions of the brain were subjected to very different levels of DNA methylation [89]. These observations suggest that methylation of cdCREs is highly tissue‐specific with implications for the relevance of extrapolating distributions of DNA methylation drawn from peripheral blood to brain structures. In addition, within GAL5.1, we detected significant variation in the levels of methylation between different CpG within the enhancer suggesting specific mechanism(s), such as genomic compaction or protection by TFs binding [89]. Unfortunately, because bisulphite sequencing was used, we were unable to differentiate between 5mC and 5hmC at specific CpGs. Future studies will involve techniques such as Nanopore sequencing, that are able to sensitively differentiate between 5mC and 5hmC. Finally, we demonstrate that 5mC had a significant repressive effect on the activity of GAL5.1. When combined with our genetic analysis of the effects of SNPs within GAL5.1 [125], these findings support the hypothesis that cdCREs can act as an important nexus that functionally link genetics and environment in health and disease. For example, one of the SNPs within GAL5.1 disrupts a CpG rendering one of the genotypes of this cdCRE less susceptible to environmental effects through DNA methylation [125] (Fig. 6). Thus, understanding the roles of DNA methylation of cdCREs in health, disease and drug response, although still in its infancy, is making steady progress.

## Conclusions

Whilst most of our understanding of the evolution, structure, function and malfunction of cdCREs has come from fields as diverse as developmental biology and the neurosciences, the same general principles apply to all fields which seek to understand the role of the noncoding genome within multicellular systems. Thanks to GWAS there is a growing recognition of the importance of the role of cdCREs in producing and maintaining the differentiated multicellular interactions required for development, homeostasis and health. So far, attempts to appropriately model the biology of cdCREs using histone markers and cell cultures alone, although very valuable, have met with limited success. Thus, we argue that there is still an urgent need to study cdCREs within living organisms. CRISPR‐CAS9 and AAV technologies allow us to manipulate the genomes of model organisms much more rapidly than what was achievable in the past. Unfortunately, in parallel with our growing understanding of the roles of cdCREs in health and disease, there is also a strong lobby to reduce or to even prevent the use of *in vivo* models in research. Given the known role of cdCREs in driving and supporting multicellularity, efforts to prevent the use of *in vivo* models will only serve to hamper our ability to understand the role of cdCREs in disease susceptibility and progression. Therefore, if we want to understand the genetic and epigenetic basis of the crippling and often deadly conditions that reduce our ability to live long healthy lives, then it is essential that we acknowledge the need to understand the roles of cdCREs in supporting multicellularity through the humane but well‐regulated use of multicellular *in vivo* models as we progress towards suitable alternatives.

## Conflict of interest

The authors declare no conflict of interest.

## Author contributions

All authors contributed to the writing and preparation of the manuscript and figures.
